# Utilisation of an operative difficulty grading scale for laparoscopic cholecystectomy

**DOI:** 10.1007/s00464-018-6281-2

**Published:** 2018-06-28

**Authors:** Ewen A. Griffiths, James Hodson, Ravi S. Vohra, Paul Marriott, the CholeS Study Group, Tarek Katbeh, Samer Zino, Ahmad H. M. Nassar, West Midlands Research Collaborative

**Affiliations:** 1 Department of Upper Gastrointestinal Surgery, University Hospitals Birmingham NHS Foundation Trust, Birmingham, UK; 2 Institute of Cancer and Genomic Sciences, College of Medical and Dental Sciences, University of Birmingham, Birmingham, UK; 3 Institute of Translational Medicine, University Hospitals Birmingham NHS Foundation Trust, Birmingham, UK; 4 Trent Oesophago-Gastric Unit, Nottingham University Hospitals NHS Trust, Nottingham, UK; 5 West Midlands Research Collaborative, Academic Department of Surgery, Birmingham University, Birmingham, UK; 6 Department of Surgery, Warwick Hospital, Lakin Rd, Warwick, UK; 7 Department of Surgery, University Hospital Monklands, Lanarkshire, Scotland, UK

**Keywords:** Surgery, Laparoscopic, Cholecystectomy, Operative difficulty, Difficulty grading

## Abstract

**Background:**

A reliable system for grading operative difficulty of laparoscopic cholecystectomy would standardise description of findings and reporting of outcomes. The aim of this study was to validate a difficulty grading system (Nassar scale), testing its applicability and consistency in two large prospective datasets.

**Methods:**

Patient and disease-related variables and 30-day outcomes were identified in two prospective cholecystectomy databases: the multi-centre prospective cohort of 8820 patients from the recent CholeS Study and the single-surgeon series containing 4089 patients. Operative data and patient outcomes were correlated with Nassar operative difficultly scale, using Kendall’s tau for dichotomous variables, or Jonckheere–Terpstra tests for continuous variables. A ROC curve analysis was performed, to quantify the predictive accuracy of the scale for each outcome, with continuous outcomes dichotomised, prior to analysis.

**Results:**

A higher operative difficulty grade was consistently associated with worse outcomes for the patients in both the reference and CholeS cohorts. The median length of stay increased from 0 to 4 days, and the 30-day complication rate from 7.6 to 24.4% as the difficulty grade increased from 1 to 4/5 (both *p* < 0.001). In the CholeS cohort, a higher difficulty grade was found to be most strongly associated with conversion to open and 30-day mortality (AUROC = 0.903, 0.822, respectively). On multivariable analysis, the Nassar operative difficultly scale was found to be a significant independent predictor of operative duration, conversion to open surgery, 30-day complications and 30-day reintervention (all *p* < 0.001).

**Conclusion:**

We have shown that an operative difficulty scale can standardise the description of operative findings by multiple grades of surgeons to facilitate audit, training assessment and research. It provides a tool for reporting operative findings, disease severity and technical difficulty and can be utilised in future research to reliably compare outcomes according to case mix and intra-operative difficulty.

**Electronic supplementary material:**

The online version contains supplementary material available at (10.1007/s00464-018-6281-2).

Laparoscopic cholecystectomy is a common operation which may vary in operative difficulty. For example, it can be a routine operation comfortably performed by a training grade surgeon (with appropriate supervision) but, at its most difficult, can tax even the most experienced specialist surgeon. It is therefore surprising that very few intra-operative difficulty scores have been published and none are widely used in clinical practice [1–3]. Moreover, none have been utilised in a large multi-centre study. The majority of previous scores use a combination of pre-operative and operative data and were produced in studies that were limited by retrospective data, small sample sizes and lack of external validation [1, 4–6]. Being able to stratify intra-operative difficulty with a simple scale of operative difficulty would have the advantages of assisting in intra-operative strategy and planning, allowing comparison across different research studies, facilitating risk adjustment for surgical outcomes and providing an aid in training surgeons and monitoring of training progression.

The Nassar operative difficulty scale is a simple 4-point scale published in 1995 and has been used in a prospective single-surgeon series which included data from 4089 patients between February 1992 and July 2014. The aim of this study was to report the utilisation of this operative grading system for laparoscopic cholecystectomy using data collected from the recent multi-centre CholeS study [7–11] and assess the grading system’s clinical utility in its association with outcome data.

## Patients and methods

For this study two large, prospective datasets containing patients treated with cholecystectomy were used.

### Reference dataset

This database started in 1992 and includes all cases managed by a single-consultant Upper GI Surgeon (AHM Nassar) in four hospitals over 22 years. The database was registered as a clinical audit in each hospital and did not require specific IRB approval. A difficulty grade was prospectively recorded for each cholecystectomy. Strict follow-up was conducted and recorded, including any complications, readmissions or 30-day reinterventions as well as outpatient review at 2–3 months. The follow-up protocol for the later part of the series (1995–2014) included 3763 cholecystectomies at two hospitals with one follow-up appointment for all laparoscopic cholecystectomies and further annual reviews of all bile duct explorations (819 cases). Follow-up included a review of the complications, readmissions and reinterventions, with emphasis on retained or recurrent stones following bile duct explorations. This is a referral firm receiving, by protocol, the majority of emergency biliary admissions and almost all patients with suspected bile duct stones admitted to the hospital. The practice includes a high rate of single-admission operations with minimal delayed operations. Higher than average rates of intra-operative cholangiography and CBD explorations were carried out, compared to normal surgical practice. Previous publications arising from this dataset and the methodology used for data collection have been published [12–16].

### CholeS dataset

The CholeS study was a multi-centre, prospective population-based cohort study of variation and outcomes of cholecystectomy [8, 9]. The protocol did not require research registration as anonymous, and observational data were collected. This was confirmed by the online NRES decision tool (http://www.hra-decisiontools.org.uk/research/) and further supported by written confirmation and advice from the Research and Development Director at University Hospitals Birmingham NHS Foundation Trust, UK. The study was registered as a ‘clinical audit’ or ‘service evaluation’ at each participating hospital under the supervision of a named senior investigator (consultant surgeon).

Data were collected from 8820 patients who underwent laparoscopic cholecystectomy in 166 hospitals across the UK, during a 2-month period from March to April 2014, and have been found to be 99.2% accurate by independent data validation. Pre-operative variables included patient demographics, indications for surgery, ASA grade, admission type, ultrasound findings and pre-operative endoscopic retrograde cholangiopancreatography (ERCP). The CholeS study protocol has been published previously [11]. The definitions of operative and outcomes parameters were similar in both studies. The duration of surgery was calculated from time (minutes) of skin incision to end of skin closure. 30-day follow-up was obtained for all patients and included rates of morbidity and mortality. All cause 30-day morbidity included bile leak, bile duct injury, wound infection, intra-abdominal collection, pancreatitis, bile duct stones, as well as non-surgical complications such as cardiac, respiratory, urinary and other complications. Bile duct injury was defined as any injury to the main biliary tree and was classified using the Stewart–Way classification [17]. Bile leak was defined using a standardised definition from the International Study Group of Liver Surgery [18].

### Nassar difficultly grading scale

In both datasets, the operative data were gathered prospectively, and surgeons were asked to grade the difficulty of the procedure using the Nassar scale (grades 1–4) [3]. This scale was published in 1995 and graded operative findings from the gallbladder, cystic pedicle and associated adhesions. The scale is as follows:

**Grade 1**:

*Gallbladder*—floppy, non-adherent

*Cystic pedicle*—thin and clear

*Adhesions*—Simple up to the neck/Hartmann’s pouch

**Grade 2**:

*Gallbladder*—Mucocele, Packed with stones

*Cystic pedicle*—Fat laden

*Adhesions*—Simple up to the body

**Grade 3**:

*Gallbladder*—Deep fossa, Acute cholecystitis, Contracted, Fibrosis, Hartmans adherent to CBD, Impaction

*Cystic pedicle*—Abnormal anatomy or cystic duct—short, dilated or obscured

*Adhesions*—Dense up to fundus; Involving hepatic flexure or duodenum

**Grade 4**:

*Gallbladder*—Completely obscured, Empyema, Gangrene, Mass

*Cystic pedicle*—Impossible to clarify

*Adhesions*—Dense, fibrosis, wrapping the gallbladder, Duodenum or hepatic flexure difficult to separate

The grading system is designed to be used as an overall summary of the operative conditions found, and the worst factor found in the individual aspect of either the ‘Gallbladder’, ‘Cystic Pedicle’ or ‘Adhesions’ should be used to define the final overall grade.

**Fig. 1:**
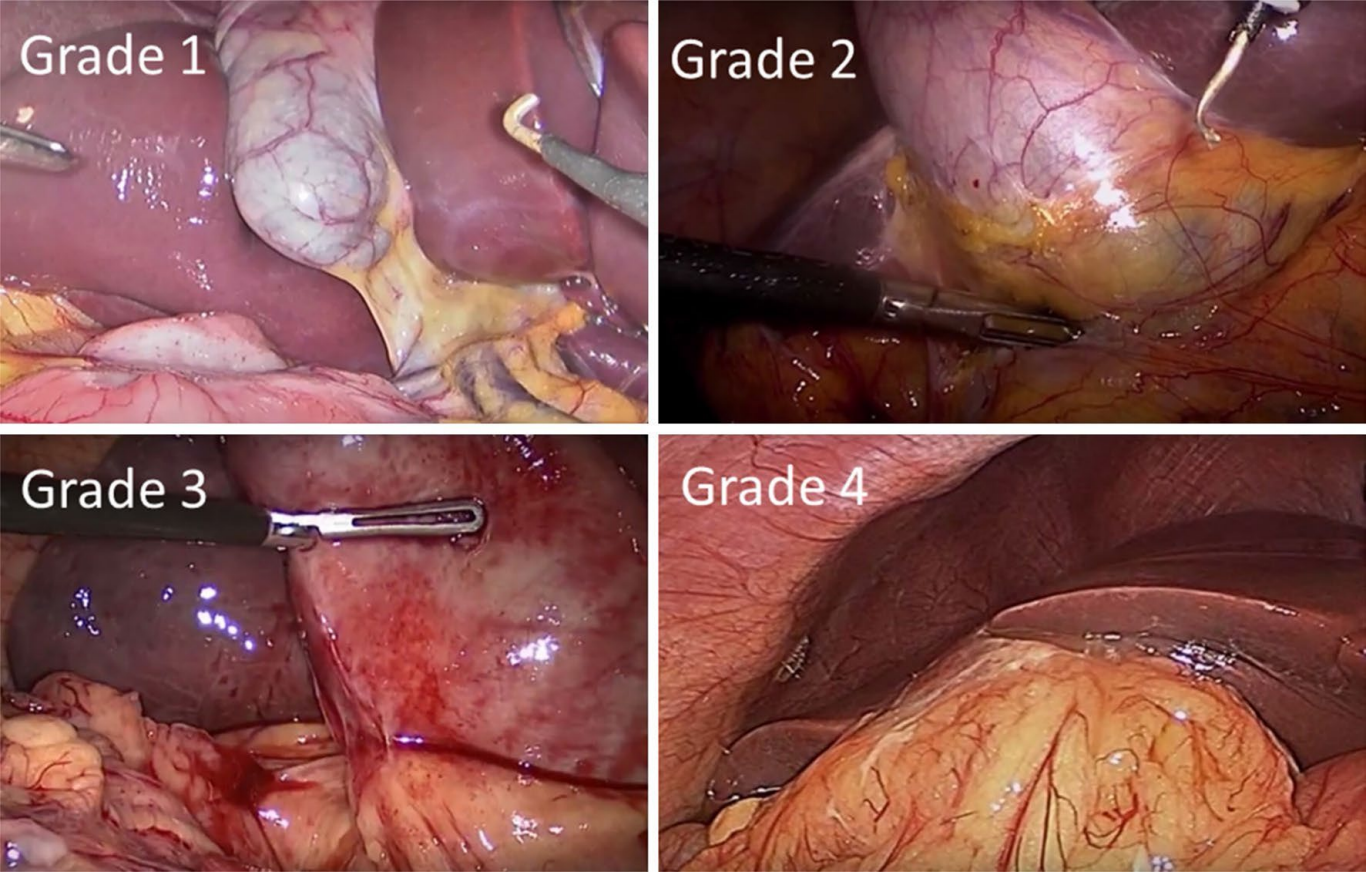
Laparoscopic photos of each Nassar operative difficulty grade. Intra-operative laparoscopic images of the Nassar operative difficulty grades are shown

Figure 1 illustrates laparoscopic images of each of the Nassar operative difficulties.

Although the difficultly scale was modified in 1996 in the reference cohort to include a Grade 5 (which was defined as the presence of either Mirizzi type 2 or higher, cholecysto-cutaneous, cholecysto-duodenal or cholecysto-colic fistula), these were combined with Grade 4 for the analysis, in order to be comparable to the scale used in the CholeS dataset and the original publication. Less than 1% of patients in the reference database had a Grade 5 operative difficulty.

### Statistical methods

Initially, a range of factors and patient outcomes were compared between the two cohorts. Continuous variables were assessed for normality, prior to the analysis. Normally distributed variables were reported as mean ± standard deviation (SD), with *p* values from independent samples *t* tests. Medians and interquartile ranges (IQRs) and Mann–Whitney tests were used where the normality assumption was not met. Nominal variables were compared using Fisher’s exact tests, with Kendall’s tau used for ordinal variables.

Patient outcomes were then correlated with Nassar operative difficultly scale, using Kendall’s tau for dichotomous variables, or Jonckheere–Terpstra tests for continuous variables. A ROC curve analysis was then performed, to quantify the predictive accuracy of the Nassar operative difficultly scale for each of the outcomes, with continuous outcomes dichotomised, prior to analysis.

The four key outcomes were then selected (conversion to open, the duration of surgery and both complications and reinterventions within 30 days), and analysed in further detail. Initially, these outcomes were compared across a range of factors, using Fisher’s exact test or Mann–Whitney/Kruskal–Wallis tests, as applicable. Multivariable analyses were then performed, to identify combinations of factors that were independently predictive of the outcomes. Binary logistic regression models were used, with variable selection by a backwards stepwise approach.

All analyses were performed using IBM SPSS 22 (IBM Corp. Armonk, NY). Patients with missing data were excluded on a test by test basis and *p* < 0.05 was deemed to be indicative of statistical significance throughout.

## Results

### Demographics

A total of 4089 cases were included in the reference cohort and 8755 operations in the CholeS cohort. Data were complete in at least 95% of cases in each of the factors considered for both of the cohorts, with the exception of ASA (85%) and length of stay (51%) in the reference cohort. Patient demographics, operative factors and outcomes are compared between the cohorts in Table 1. This identified a range of differences between the two cohorts. For example, higher percentage of emergency admissions was found in the reference dataset due to the nature of the referral protocol of the acute biliary service. However, there were higher rates of cholecystitis and thick-walled gallbladders in the CholeS dataset. MRCP and ERCP were used less frequently in the reference dataset, due to a preference to perform intra-operative cholangiography. There was also a higher rate of CBD exploration, drain use and length of hospital stay in the reference cohort.

**Table 1:** Comparison of demographic, pre-operative factors, operative factors and patient outcomes between the cohorts

	Reference dataset		CholeS dataset		*p* value
	Valid *N*	Statistic	Valid *N*	Valid *N*	
Demographics					
Age	4030	50.4 ± 15.9	8748	51.0 ± 16.5	0.067
Gender (male)	4068	981 (24.1%)	8755	2281 (26.1%)	**0.020**
Diagnosis	4048		8749		< **0.001**
CBD stone		598 (14.8%)		557 (6.4%)	
Cholecystitis		674 (16.7%)		2530 (28.9%)	
Colic		2502 (61.8%)		4816 (55.0%)	
Pancreatitis		274 (6.8%)		846 (9.7%)	
Admission type	4027		8755		< **0.001**
Delay		861 (21.4%)		3247 (37.1%)	
Elective		1883 (46.8%)		4117 (47.0%)	
Emergency		1283 (31.9%)		1391 (15.9%)	
Pre-operative investigations					
USS	4089	3769 (92.2%)	8744	8409 (96.2%)	< **0.001**
Thick-walled gallbladder	4089	565 (13.8%)	8548	2800 (32.8%)	< **0.001**
CBD dilation	4089	639 (15.6%)	8552	1351 (15.8%)	0.814
CT	4089	66 (1.6%)	8654	1257 (14.5%)	< **0.001**
MRCP	4089	173 (4.2%)	8662	2264 (26.1%)	< **0.001**
ERCP	4089	143 (3.5%)	8650	931 (10.8%)	< **0.001**
Peri-operative factors					
Nassar scale	4035		8680		< 0.001*
1		1359 (33.7%)		3524 (40.6%)	
2		1260 (31.2%)		2608 (30.0%)	
3		802 (19.9%)		1769 (20.4%)	
4/5		614 (15.2%)		779 (9.0%)	
ASA	3496		8681		0.501*
1		1455 (41.6%)		3354 (38.6%)	
2		1570 (44.9%)		4436 (51.1%)	
3		464 (13.3%)		869 (10.0%)	
4/5		7 (0.2%)		22 (0.3%)	
Duration of surgery (min)	4054	60 (45–85)	8550	62 (47–90)	< **0.001**
Bile spilt	4089	199 (4.9%)	8690	2343 (27.0%)	< **0.001**
Stones spilt	4089	102 (2.5%)	8677	830 (9.6%)	< **0.001**
Bleeding	4089	16 (0.4%)	8677	739 (8.5%)	< **0.001**
Bowel injury	4089	3 (0.1%)	8674	48 (0.6%)	< **0.001**
CBD injury	4089	2 (0.0%)	8615	23 (0.3%)	**0.009**
Post-surgical drain	3928	2039 (51.9%)	8735	1609 (18.4%)	< **0.001**
Converted to open	4015	27 (0.7%)	8755	297 (3.4%)	< **0.001**
Cholangiography	4064	3635 (89.4%)	8751	1052 (12.0%)	< **0.001**
CBD explored	4088	874 (21.4%)	8745	256 (2.9%)	< **0.001**
Patient outcomes					
Total length of stay (days)	2077	3 (1–6)	8719	1 (0–2)	< **0.001**
30-day readmissions	4089	93 (2.3%)	8755	618 (7.1%)	< **0.001**
30-day complications	4089	297 (7.3%)	8755	937 (10.7%)	< **0.001**
30-day reintervention	4089	62 (1.5%)	8755	762 (8.7%)	< **0.001**
30-day mortality	4089	4 (0.1%)	8755	10 (0.1%)	1.000

Data reported as *N* (%), with *p* values from Fisher’s exact tests, or as mean ± SD, with *p* values from *t* tests, unless stated otherwise. Valid *N* = the number of patients for whom data were available.

*p value from Kendall’s tau, as the factor was ordinal

Bold *p* values are significant at *p* < 0.05

### Associations with Nassar operative difficulty scale

Associations between the Nassar operative difficulty scale, operative factors and patient outcomes were then examined (Table 2). Due to the previously identified differences between the reference and CholeS cohorts, the two datasets were analysed separately.

**Table 2:** Associations between Nassar operative difficulty scale and operative factors and patient outcomes

	Nassar operative difficulty scale				*p* value
	1	2	3	4/5	
Peri-operative factors					
Duration of surgery (min)					
CholeS	55 (40–70)	60 (50–83)	80 (60–105)	110 (80–145)	< **0.001**
Reference	45 (35–60)	60 (45–75)	73 (55–95)	110 (85–150)	< **0.001**
Bile spilt					
CholeS	528 (15.1%)	665 (25.7%)	717 (40.7%)	422 (54.5%)	< **0.001**
Reference	48 (3.5%)	54 (4.3%)	55 (6.9%)	42 (6.8%)	< **0.001**
Stones spilt					
CholeS	85 (2.4%)	171 (6.6%)	316 (18.0%)	254 (32.9%)	< **0.001**
Reference	9 (0.7%)	27 (2.1%)	31 (3.9%)	35 (5.7%)	< **0.001**
Bleeding					
CholeS	114 (3.3%)	217 (8.4%)	221 (12.6%)	183 (23.7%)	< **0.001**
Reference	5 (0.4%)	3 (0.2%)	3 (0.4%)	5 (0.8%)	0.352
Bowel injury					
CholeS	6 (0.2%)	13 (0.5%)	9 (0.5%)	20 (2.6%)	< **0.001**
Reference	0 (0.0%)	0 (0.0%)	1 (0.1%)	2 (0.3%)	0.091
CBD injury					
CholeS	1 (0.0%)	5 (0.2%)	3 (0.2%)	13 (1.7%)	< **0.001**
Reference	0 (0.0%)	1 (0.1%)	0 (0.0%)	1 (0.2%)	0.325
Post-surgical drain					
CholeS	205 (5.8%)	280 (10.7%)	574 (32.5%)	539 (69.3%)	< **0.001**
Reference	328 (25.1%)	553 (45.5%)	566 (73.6%)	568 (95.9%)	< **0.001**
Converted to open					
CholeS	7 (0.2%)	12 (0.5%)	65 (3.7%)	212 (27.2%)	< **0.001**
Reference	2 (0.1%)	1 (0.1%)	4 (0.5%)	20 (3.4%)	< **0.001**
Patient outcomes					
Total length of stay (days)					
CholeS	0 (0–1)	1 (0–1)	1 (0–3)	4 (1–8)	< **0.001**
Reference	2 (1–4)	3 (1–5)	4 (2–7)	6 (4–9)	< **0.001**
30-day readmissions					
CholeS	226 (6.4%)	180 (6.9%)	134 (7.6%)	65 (8.3%)	**0.035**
Reference	19 (1.4%)	31 (2.5%)	20 (2.5%)	22 (3.6%)	**0.003**
30-day complications					
CholeS	267 (7.6%)	258 (9.9%)	204 (11.5%)	190 (24.4%)	< **0.001**
Reference	65 (4.8%)	74 (5.9%)	80 (10.0%)	78 (12.7%)	< **0.001**
30-day reintervention					
CholeS	230 (6.5%)	202 (7.7%)	160 (9.0%)	157 (20.2%)	< **0.001**
Reference	9 (0.7%)	14 (1.1%)	16 (2.0%)	22 (3.6%)	< **0.001**
30-day mortality					
CholeS	1 (0.0%)	0 (0.0%)	4 (0.2%)	5 (0.6%)	**0.009**
Reference	0 (0.0%)	0 (0.0%)	1 (0.1%)	3 (0.5%)	**0.050**

Data reported as *N* (%), with *p* values from Kendall’s Tau, or as median (IQR), with *p* values from Jonckheere–Terpstra tests, as applicable

Bold *p* values are significant at *p* < 0.05

**Fig. 2:**
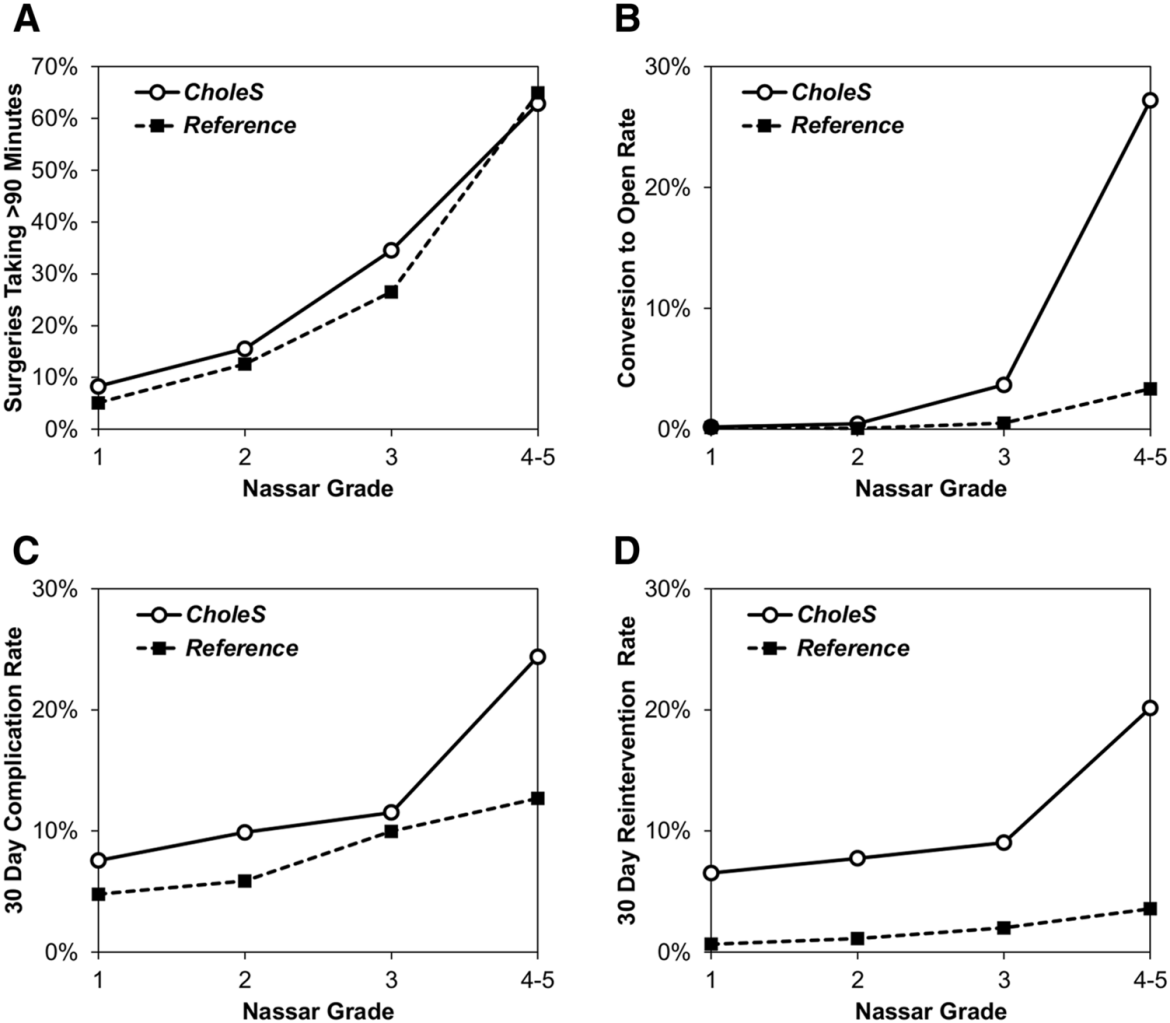
Rates of key factors and outcomes by Nassar operative difficulty scale in the two cohorts

Increasing Nassar operative difficulty scale was consistently associated with significantly worse outcomes for the patients in the CholeS cohort. For example, the median length of stay increased from 0 to 4 days, and the 30-day complication rate from 7.6 to 24.4% as the Nassar scale increased from 1 to 4/5 (both *p* < 0.001). Similar outcomes were observed in the reference cohort, although not all of the associations reached significance in both cohorts. However, the outcomes where no significant association with the Nassar scale was detected were rare events where statistical power would have been too low to identify a trend. For example, CBD injury, which was not found to be significantly associated with the Nassar scale (*p* = 0.325) in the reference cohort, only occurred in *n* = 2 patients. Selected outcomes are also reported graphically in Fig. 2.

The relationships between Nassar scale and both operative factors and patient outcomes were also considered in a ROC curve analysis (Table 3). Surgical duration and length of stay were dichotomised, using cut-off values of > 90 min and > 5 days, respectively. In the CholeS cohort, the Nassar scale was found to be most strongly associated with conversion to open and 30-day mortality (AUROC = 0.903, 0.822, respectively).

**Table 3:** ROC curve analysis of the associations between Nassar operative difficultly scale and operative factors and patient outcomes

	AUROC (SE)	
	CholeS	Reference
Peri-operative factors		
Duration of surgery > 90 min	0.743 (0.007)	0.797 (0.009)
Bile spilt	0.673 (0.007)	0.580 (0.021)
Stones spilt	0.764 (0.009)	0.695 (0.024)
Bleeding	0.693 (0.010)	0.574 (0.080)
Bowel injury	0.727 (0.038)	0.866 (0.056)
CBD injury	0.811 (0.046)	0.709 (0.160)
Post-surgical drain	0.789 (0.007)	0.777 (0.007)
Converted to open	0.903 (0.009)	0.830 (0.042)
Patient outcomes		
Total length of stay > 5 days	0.698 (0.010)	0.692 (0.014)
30-day readmissions	0.525 (0.012)	0.586 (0.029)
30-day complications	0.603 (0.010)	0.612 (0.017)
30-day reintervention	0.593 (0.011)	0.670 (0.035)
30-day mortality	0.822 (0.071)	0.880 (0.045)

Data reported as area under ROC curves (AUROC) and standard errors (SE)

### Other predictors of patient outcome

Four clinically relevant outcomes, namely conversion to open, the duration of surgery and both complications and reinterventions within 30 days, were then considered in more detail. Initially, a range of pre-operative factors were compared to each of these outcomes in the CholeS cohort (Table 4). All considered outcomes were worse in patients with increasing age, ASA grade, male gender, non-elective admissions, the use of pre-operative CT/MRCP/ERCP, CBD dilation on pre-operative imaging, those without USS and for diagnoses of CBD stones or Cholecystitis. Increasing BMI was also associated with a significantly increased surgical duration as did thick-walled gallbladders and were more likely to be converted to open.

**Table 4:** Associations between pre-operative factors and selected outcomes in the CholeS cohort

	*N*	Duration of surgery	Converted to open	30-day complications	30-day reintervention
Age (years)		*p* < 0.001	*p* < 0.001	*p* < 0.001	*p* < 0.001
< 40	2346	60 (45–80)	20 (0.9%)	233 (9.9%)	212 (9.0%)
40–49	1678	61 (46–90)	41 (2.4%)	156 (9.3%)	130 (7.7%)
50–64	2601	65 (49–90)	113 (4.3%)	245 (9.4%)	194 (7.5%)
65+	2123	69 (50–95)	123 (5.8%)	302 (14.2%)	226 (10.6%)
Gender		*p* < 0.001	*p* < 0.001	*p* = 0.005	*p* = 0.154
Female	6474	60 (45–86)	153 (2.4%)	657 (10.1%)	547 (8.4%)
Male	2281	70 (51–96)	144 (6.3%)	280 (12.3%)	215 (9.4%)
BMI		*p* < 0.001	*p* = 0.834	*p* = 0.905	*p* = 0.937
≤ 30	4733	60 (45–88)	158 (3.3%)	492 (10.4%)	406 (8.6%)
31–35	2046	65 (50–90)	71 (3.5%)	211 (10.3%)	170 (8.3%)
> 35	1573	68 (50–90)	57 (3.6%)	169 (10.7%)	135 (8.6%)
Diagnosis		*p* < 0.001	*p* < 0.001	*p* < 0.001	*p* < 0.001
CBD stone	557	75 (55–118)	52 (9.3%)	84 (15.1%)	67 (12.0%)
Cholecystitis	2530	75 (56–104)	165 (6.5%)	332 (13.1%)	262 (10.4%)
Colic	4816	60 (45–77)	62 (1.3%)	406 (8.4%)	331 (6.9%)
Pancreatitis	846	69 (50–90)	18 (2.1%)	114 (13.5%)	102 (12.1%)
Admission type		*p* < 0.001	*p* < 0.001	*p* < 0.001	*p* < 0.001
Elective	4117	60 (45–78)	68 (1.7%)	316 (7.7%)	240 (5.8%)
Delay	3247	65 (50–90)	152 (4.7%)	412 (12.7%)	335 (10.3%)
Emergency	1391	80 (60–110)	77 (5.5%)	209 (15.0%)	187 (13.4%)
ASA		*p* < 0.001	*p* < 0.001	*p* < 0.001	*p* < 0.001
1	3354	60 (45–80)	43 (1.3%)	227 (8.3%)	243 (7.2%)
2	4436	65 (50–90)	171 (3.9%)	448 (11.0%)	391 (8.8%)
3	869	74 (53–101)	77 (8.9%)	153 (17.6%)	118 (13.6%)
4–5	22	82 (45–120)	1 (4.5%)	7 (31.8%)	4 (18.2%)
USS		*p* < 0.001	*p* < 0.001	*p* < 0.001	*p* = 0.023
No	335	75 (58–110)	24 (7.2%)	57 (17.0%)	41 (12.2%)
Yes	8409	61 (46–90)	273 (3.2%)	878 (10.4%)	720 (8.6%)
Thick-walled gallbladder		*p* < 0.001	*p* < 0.001	*p* = 0.144	*p* = 0.175
No	5748	60 (45–80)	117 (2.0%)	589 (10.2%)	477 (8.3%)
Yes	2800	72 (54–100)	166 (5.9%)	316 (11.3%)	257 (9.2%)
CBD dilation		*p* < 0.001	*p* < 0.001	*p* < 0.001	*p* < 0.001
No	7201	60 (45–87)	197 (2.7%)	708 (9.8%)	561 (7.8%)
Yes	1351	72 (53–105)	85 (6.3%)	196 (14.5%)	177 (13.1%)
CT		*p* < 0.001	*p* < 0.001	*p* < 0.001	*p* < 0.001
No	7397	60 (45–90)	203 (2.7%)	722 (9.8%)	596 (8.1%)
Yes	1257	72 (55–100)	89 (7.1%)	206 (16.4%)	161 (12.8%)
MRCP		*p* < 0.001	*p* < 0.001	*p* < 0.001	*p* < 0.001
No	6398	60 (45–89)	176 (2.8%)	627 (9.8%)	510 (8.0%)
Yes	2264	70 (51–92)	115 (5.1%)	301 (13.3%)	246 (10.9%)
ERCP		*p* < 0.001	*p* < 0.001	*p* < 0.001	*p* < 0.001
No	7719	61 (46–90)	215 (2.8%)	780 (10.1%)	635 (8.2%)
Yes	931	75 (55–105)	76 (8.2%)	147 (15.8%)	121 (13.0%)

Dichotomous outcomes are reported as *N* (%), with *p* values from Fisher’s exact test. Duration of surgery is reported as median (IQR), with *p* values from Mann–Whitney and Kruskal–Wallis tests for comparisons across two and more than two groups, respectively

Bold *p* values are significant at *p* < 0.05

### Multivariable analysis

A set of multivariable analyses were then performed, to assess whether there was an independent association between the Nassar scale and patient outcome, after accounting for other factors previously identified as being associated with patient outcome (Table 5). This found a range of factors that were independently associated with the patient outcomes being considered, including increasing patient age, non-elective admissions and increasing ASA grade. After accounting for these factors, the Nassar scale remained a significant predictor of all four outcomes (all *p* < 0.001), with odds ratios for Nassar grade 4–5 versus 1 of 13.5, 115.6, 3.18 and 2.91 for surgical duration of more than 90 min, conversion to open and 30-day complication and reintervention rates, respectively.

**Table 5:** Multivariable analyses of binary outcomes in the CholeS dataset

	Surgery > 90 min		Converted to open		30-day complications		30-day reintervention	
	OR (95% CI)	*p* value	OR (95% CI)	*p* value	OR (95% CI)	*p* value	OR (95% CI)	*p* value
Nassar grade		< **0.001**		< **0.001**		< **0.001**		< **0.001**
1	1	–	1	–	1	–	1	–
2	1.81 (1.52–2.15)	< **0.001**	2.07 (0.81–5.26)	0.129	1.27 (1.05–1.55)	**0.014**	1.10 (0.89–1.36)	0.370
3	4.47 (3.76–5.31)	< **0.001**	12.26 (5.51–27.29)	< **0.001**	1.41 (1.13–1.75)	**0.002**	1.22 (0.97–1.55)	0.094
4–5	14.24 (11.44–17.74)	< **0.001**	115.6 (52.9–252.9)	< **0.001**	3.18 (2.48–4.08)	< **0.001**	2.91 (2.23–3.80)	< **0.001**
Age (years)		**0.035**		0.119		**0.002**		**0.001**
< 40	1	–	1	–	1	–	1	–
40–49	1.26 (1.04–1.53)	**0.020**	1.67 (0.91–3.06)	0.096	0.81 (0.64–1.02)	0.070	0.77 (0.60–0.99)	**0.039**
50–64	1.27 (1.06–1.51)	**0.008**	1.94 (1.13–3.32)	0.016	0.69 (0.55–0.86)	< **0.001**	0.62 (0.49–0.79)	< **0.001**
65+	1.23 (1.02–1.47)	**0.028**	1.68 (0.97–2.92)	0.067	0.94 (0.75–1.17)	0.576	0.79 (0.62–1.01)	0.064
BMI		< **0.001**		*NS*		*NS*		*NS*
≤ 30	1	–	–	–	–	–	–	–
31–35	1.11 (0.95–1.28)	0.182	–	–	–	–	–	–
> 35	1.42 (1.21–1.66)	< **0.001**	–	–	–	–	–	–
Diagnosis		< **0.001**		< **0.001**		*NS*		*NS*
CBD stone	1	–	1	–	–	–	–	–
Cholecystitis	0.69 (0.52–0.91)	**0.009**	0.45 (0.30–0.68)	< **0.001**	–	–	–	–
Colic	0.55 (0.41–0.74)	< **0.001**	0.37 (0.23–0.59)	< **0.001**	–	–	–	–
Pancreatitis	0.74 (0.55–1.00)	**0.047**	0.28 (0.14–0.55)	< **0.001**	–	–	–	–
Admission type		< **0.001**		*NS*		< **0.001**		< **0.001**
Elective	1	–	–	–	1	–	1	–
Delay	1.18 (1.00–1.40)	**0.048**	–	–	1.47 (1.22–1.75)	< **0.001**	1.48 (1.21–1.81)	< **0.001**
Emergency	1.88 (1.55–2.29)	< **0.001**	–	–	1.68 (1.35–2.09)	< **0.001**	2.02 (1.60–2.56)	< **0.001**
ASA		*NS*		**0.004**		< **0.001**		**0.023**
1	–	–	1	–	1	–	1	–
2	–	–	1.68 (1.12–2.50)	**0.011**	1.19 (0.99–1.42)	0.061	1.16 (0.95–1.41)	0.139
3	–	–	2.38 (1.48–3.82)	< **0.001**	1.66 (1.28–2.15)	< **0.001**	1.54 (1.16–2.05)	**0.003**
4–5	–	–	0.67 (0.08–5.60)	0.711	3.37 (1.28–8.85)	**0.014**	1.83 (0.58–5.79)	0.304
CBD dilation	1.55 (1.30–1.84)	< **0.001**	–	*NS*	1.29 (1.07–1.55)	**0.008**	1.46 (1.20–1.78)	< **0.001**
CT	–	*NS*	–	*NS*	1.28 (1.05–1.56)	**0.014**	1.21 (0.97–1.50)	0.084
ERCP	0.82 (0.65–1.04)	0.098	–	*NS*	–	*NS*	–	*NS*
Thick-walled gallbladder	–	*NS*	–	*NS*	0.69 (0.58–0.82)	< **0.001**	0.72 (0.60–0.87)	< **0.001**

Results are from multivariable binary logistic regression models, using a backwards stepwise variable selection approach, and with all factors in Supplementary Table 3 considered for inclusion

*NS* not selected for inclusion in the final model by the stepwise procedure

Bold *p* values are significant at *p* < 0.05

## Discussion

We have shown that a simple scale of operative difficulty in laparoscopic cholecystectomy can be easily applied to patients across two separate large cohort databases. Despite the baseline differences in these datasets, the operative difficultly score remained highly clinical relevant. It would seem that, when given the concept and the criteria of the difficulty grading, a large number of surgeons will consistently classify cholecystectomies in a similar manner. We have also shown that a higher difficulty grade has strong clinical relevance, being associated with worse clinical outcomes, and that this association is independent of other factors on multivariable analysis.

Due to the variability of operative findings, laparoscopic cholecystectomy is one of the most unpredictable operations in general surgery, This can be due to anatomical reasons, but is mainly due to the effect of cholecystitis and fibrosis on the dissection planes in Calot’s triangle. Publications reporting surgical outcomes following cholecystectomy are difficult to compare, as currently no grading or scoring system is consistently used to document operative findings. This was why the CholeS study incorporated the Nassar intra-operative difficulty grading method [3] into its protocol [11] and asked participating surgeons to view online videos of varying Nassar grades prior to study commencement. Although a number of important clinical applications of the Nassar grading system have been reported, the scale has yet to be evaluated and validated in large cohorts of patients such as the present study. Previous publications using the scale addressed the optimisation of the management of complicated gallstone disease [19, 20] and the suitability of certain cases for single-port laparoscopic cholecystectomy versus four-port laparoscopic cholecystectomy [21].

Very few intra-operative difficultly scores for use in cholecystectomy have been published [1, 3–6] (Table 6). Sugrue et al. have developed a scoring system using operative findings, incorporating the appearance of the gallbladder, presence of gallbladder distension, ease of access, potential biliary complications and time taken to identify cystic duct and artery [1]. However, no clinical outcome data were presented in this paper and no validation of its clinical usefulness was performed.

**Table 6:** Available intra-operative difficulty scores for cholecystectomy

Nassar scale (present paper)	
Grade 1	
*Gallbladder*—floppy, non-adherent	
*Cystic pedicle*—thin and clear	
*Adhesions*—Simple up to the neck/Hartmann’s pouch	
Grade 2	
*Gallbladder*—Mucocele, Packed with stones	
*Cystic pedicle*—Fat laden	
*Adhesions*—Simple up to the body	
Grade 3	
*Gallbladder*—Deep fossa, Acute cholecystitis, Contracted, Fibrosis, Hartmans adherent to CBD, Impaction	
*Cystic pedicle*—Abnormal anatomy or cystic duct—short, dilated or obscured	
*Adhesions*—Dense up to fundus; Involving hepatic flexure or duodenum	
Grade 4	
*Gallbladder*—Completely obscured, Empyema, Gangrene, Mass	
*Cystic pedicle*—Impossible to clarify	
*Adhesions*—Dense, fibrosis, wrapping the gallbladder, Duodenum or hepatic flexure difficult to separate	
Correlation with outcome data available?	
Yes, this paper reports outcome from a single-surgeon series of 4089 patients and validation in a large multi-centre prospective cohort of 8820. Increasingly difficulty associated with worse clinical outcomes including 30-day complications, reintervention, length of stay and conversion to open surgery. Independent on multivariate analysis	

Cuschieri scale [2, 22]	
Grade 1: easy/uncomplicated cholecystectomy	
Grade 2: medium difficulty, for example mild cholecystitis, cystic duct or artery obscured by adhesions or fatty tissue; mucocele may be present	
Grade 3: difficult cholecystectomy due to either gangrenous cholecystitis; shrunken fibrotic gallbladder; severe cholecystitis; subhepatic abscess formation; Hartman pouch adherent to the CHD; cases in which the cystic duct or artery are difficult or impossible to dissect; or liver cirrhosis with portal hypertension	
Grade 4: conversion to open surgery is required	
Correlation with outcome data available?	
No	

Parkland scale [24]	
Grade 1: normal gallbladder/no adhesions	
Grade 2: minor adhesions at the neck	
Grade 3: presence of ANY of the following: hyperemia, pericholecystic fluid, adhesions to the body, distended gallbladder	
Grade 4: presence of ANY of the following: Adhesions obscuring majority of gallbladder or Grade I–III with abnormal liver anatomy, intrahepatic gallbladder, or impacted stone (Mirizzi)	
Grade 5: presence of ANY of the following: Perforation, necrosis, inability to visualise the gallbladder due to adhesions	
Correlation with outcome data available?	
Outcome data available for 50 patients showing increasing severity were associated with longer operating times, length of stay and post-operative bile leaks	

Sugrue et al. [1]	
Gallbladder appearance	Points
Adhesions < 50% of GB	1
Adhesions burying GB	3
Distension/contraction	
Distended GB (or contracted shrivelled GB)	1
Unable to grasp with atraumatic laparoscopic forceps	1
Stone ≥ 1 cm impacted in Hartman’s Pouch	1
Access	
BMI > 30	1
Adhesions from previous surgery limiting access	1
Severe sepsis/complications	
Bile or Pus outside GB	1
Time to identify cystic artery and duct > 90 min	1
*Total max*	*10*
Degree of difficulty	
(A) Mild < 2; (B) Moderate = 2–4; (C) Severe = 5–7; (D) Extreme = 8–10	
Correlation with outcome data available?	
No	

Cuschieri published a ‘scale of difficulty’ for laparoscopic cholecystectomy in a textbook in 1992 [22] and this was subsequently modified in a further publication in The Lancet in 1998 [2] (Table 6). However, it can be argued that with increasing skill level in laparoscopic surgery over the last 20 years, even very difficult operations can be now managed without conversion to open surgery. For example, laparoscopic “damage control” methods, including cholecystostomy, fundus first cholecystectomy and subtotal cholecystectomy, have been proposed to avoid conversion to open surgery [15, 23]. This means that the Cuschieri scale is no longer applicable in the current era. In addition, conversion to open surgery could be required in cases of fairly simple cholecystectomy due to other reasons, such as uncontrollable bleeding or iatrogenic injury. As previously reported by the CholeS study group, the threshold for conversion is likely to vary between surgeons, and may relate to several factors, such as patient related factors, surgeon’s experience and procedural difficulty [10].

A recent paper categorised intra-operative photographs of patients undergoing cholecystectomy and developed the ‘Parkland’ grading scale for cholecystectomy [24], which is broadly comparable to the Nassar operative difficulty scale. Outcome data were only presented for 50 patients, but increasing severity was associated with longer operating times, length of stay and post-operative bile leaks [24], which is in keeping with our findings.

Our future aim is to develop a risk prediction tool for intra-operative difficulty which will use pre-operative variables to predict a more difficult and taxing operation. This could then be used for the selection of patients for day-case surgery or to anticipate a difficult operation and either allow more theatre time or employ the services of a more specialist surgeon or unit.

This study has some limitations. There will be some subjectivity in the use of the operative difficultly scale between surgeons. There were some baseline clinical differences between the two datasets. The reference cohort was based on the experience of a specialist biliary surgeon that performed more 4000 laparoscopic cholecystectomies over more than 20 years, whilst the CholeS cohort was made up of over 8000 operations performed in a 2-month period by many surgeons with different types and degrees of experience. However, the fact that the Nassar operative difficulty scale remained clinically relevant in both datasets is a testament to its simplicity and clinical relevance. In contrast to other papers published on the operative difficulty of cholecystectomy [1, 22, 24], our paper used large, prospectively collected data with highly validated outcome data. Whilst the score was developed and used in a single-centre dataset with a long study duration, the validation has been performed in a dataset which includes multiple centres and high-quality external data validation.

## Conclusion

We have shown that this simple operative difficulty scale can be used by multiple grades of surgeons (including trainees and consultants) and remain highly clinically relevant. Our study demonstrated the applicability, consistency and reproducibility of the grading process. It therefore provides a tool for reporting disease and intra-operative severity and can reliably be utilised in future research to adjust outcomes according to case mix and intra-operative difficulty. The grading of operative difficulty should be collected routinely.

## Electronic supplementary material

Below is the link to the electronic supplementary material.

Supplementary material 1 (DOCX 25 KB)
