# Supplementary material for: Utilisation of an operative difficulty grading scale for laparoscopic cholecystectomy
Source: Surg Endosc. 2018 Jun 28;33(1):110–21. doi: 10.1007/s00464-018-6281-2 (PMC6336748; doi:10.1007/s00464-018-6281-2)
Supplement: Supplementary file 1 — Supplementary material 1 (DOCX 25 KB) [file 464_2018_6281_MOESM1_ESM.docx]

**CholeS Study Management Team**

Ravinder S. Vohra, Consultant Surgeon, Nottingham Oesophago-Gastric Unit, Nottingham University Hospitals NHS Foundation Trust, Hucknall Road, Nottingham, UK;

Amanda J. Kirkham, Biostatistician; Cancer Research UK Clinical Trials Unit, The University of Birmingham, Birmingham, UK;

Sandro Pasquali, Surgical trainee, Surgical Oncology Unit, Veneto Institute of Oncology IOV-IRCCS, Padova, Italy;

Paul Marriott, Surgical trainee, West Midlands Research Collaborative, Academic Department of Surgery, The University of Birmingham, Birmingham, UK;

Marianne Johnstone, Surgical trainee, West Midlands Research Collaborative, Academic Department of Surgery, The University of Birmingham, Birmingham, UK;

Philip Spreadborough, Surgical trainee, West Midlands Research Collaborative, Academic Department of Surgery, The University of Birmingham, Birmingham, UK

Derek Alderson, Emeritus Professor of Surgery, Academic Department of Surgery, The University of Birmingham, Birmingham, UK;

Ewen A. Griffiths, Consultant Surgeon, Department of Upper Gastrointestinal Surgery, University Hospitals Birmingham NHS Foundation Trust, Birmingham, UK

**CholeS Study Collaborators:**

**England** – Stephen Fenwick, Mohamed Elmasry, Quentin M Nunes, David Kennedy (Aintree University Hospital NHS Foundation Trust); Raja Basit Khan, Muhammad AS Khan (Airedale General Hospital); Conor J Magee, Steven M Jones, Denise Mason, Ciny P Parappally (Wirral University Teaching Hospital); Pawan Mathur, Michael Saunders, Sara Jamel, Samer Ul Haque, Sara Zafar (Barnet and Chase Farm Hospital); Muhammad Hanif Shiwani, Nehemiah Samuel, Farooq Dar, Andrew Jackson (Barnsley District General Hospital); Bryony Lovett, Shiva Dindyal, Hannah Winter, Ted Fletcher, Saquib Rahman (Basildon Univesity Hospital); Kevin Wheatley, Tom Nieto, Soofiyah Ayaani (Sandwell and West Birmingham Hospitals NHS Trust); Haney Youssef, Rajwinder S Nijjar, Helen Watkin, David Naumann, Sophie Emesih; Piyush B Sarmah, Kathryn Lee, Nikita Joji, Joel Lambert (Heart of England Foundation NHS Trust); Jonathan Heath, Rebecca L Teasdale, Chamindri Weerasinghe (Blackpool Teaching Hospitals NHS Foundation Trust); Paul J Needham, Hannah Welbourn, Luke Forster, David Finch (Bradford Teaching Hospitals NHS Foundation Trust); Jane M Blazeby, William Robb, Angus GK McNair, Alex Hrycaiczuk (University Hospitals Bristol NHS Trust); Alexandros Charalabopoulos, Sritharan Kadirkamanathan, Cheuk-Bong Tang, Naga VG Jayanthi, Nigel Noor (Broomfield Hospital); Brian Dobbins, Andrew J Cockbain, April Nilsen-Nunn, Jonathan de Siqueira (Calderdale and Huddersfield NHS Trust); Mike Pellen, Jonathan B Cowley, Wei-Min Ho, Victor Miu (Hull and East Yorkshire NHS Trust); Timothy J White, Kathryn A Hodgkins, Alison Kinghorn (Chesterfield Royal Hospital NHS Foundation Trust); Matthew G Tutton, Yahya A Al-Abed, Donald Menzies, Anwar Ahmad, Joanna Reed, Shabuddin Khan (Colchester Hospital University NHS Foundation Trust); David Monk, Louis J Vitone, Ghulam Murtaza, Abraham Joel (Countess of Chester NHS Foundation Trust); Stephen Brennan, David Shier, Catherine Zhang, Thusidaran Yoganathan (Croydon Health Services NHS Trust); Steven J Robinson, Iain JD McCallum, Michael J Jones, Mohammed Elsayed, Liz Tuck, John Wayman, Kate Carney (North Cumbria University Hospitals Trust); Somaiah Aroori, Kenneth B Hosie, Adam Kimble, David M Bunting, Kenneth B Hosie (Plymouth Hospitals NHS Trust); Adeshina S Fawole, Mohammed Basheer, Rajiv V Dave, Janahan Sarveswaran, Elinor Jones, Chris Kendal (Mid Yorkshire NHS Trust); Michael P Tilston, Martin Gough, Tom Wallace, Shailendra Singh, Justine Downing Katherine A Mockford, Eyad Issa, Nayab Shah, Neal Chauhan (Northern Lincolnshire and Goole NHS Foundation Trust); Timothy R Wilson, Amir Forouzanfar, Jonathan RL Wild, Emma Nofal, Catherine Bunnell, Khaliel Madbak (Doncaster and Bassetlaw Hospitals NHS Foundation Trust); Sudhindra TV Rao, Laurence Devoto, Najaf Siddiqi, Zechan Khawaja (Dorset County Hospital NHS Foundation Trust); James C Hewes, Laura Gould, Alice Chambers, Daniel Urriza Rodriguez (North Bristol NHS Trust); Gourab Sen, Stuart Robinson, Kate Carney, Francis Bartlett (Freeman Hospital); David M Rae, Thomas EJ Stevenson, Kas Sarvananthan (Frimley Park Hospital NHS Trust); Simon J Dwerryhouse, Simon M Higgs, Oliver J Old, Thomas J Hardy, Reena Shah Steve T Hornby, Ken Keogh, Lucinda Frank (Gloucestershire Hospitals NHS Trust); Musallam Al-Akash, Emma A Upchurch (Great Western Hospitals NHS Foundation Trust); Richard J Frame, Michael Hughes, Clare Jelley (Harrogate and District NHS Foundation Trust); Simon Weaver, Sudipta Roy, Toritseju O Sillo, Giorgios Galanopoulos (Wye Valley NHS Trust); Tamzin Cuming, Pedro Cunha, Salim Tayeh, Sarantos Kaptanis (Homerton University Hospital NHS Trust); Mohamed Heshaishi, Abdalla Eisawi, Michael Abayomi; Wee Sing Ngu, Katie Fleming, Dalvir S Bajwa (Tees Hospitals NHS Foundation Trust); Vivek Chitre, Kamal Aryal, Paul Ferris (Paget University Hospitals NHS Foundation Trust); Michael Silva, Simon Lammy Sarah Mohamed, Amir Khawaja, Adnan Hussain, Mudassar A Ghazanfar, Maria Irene Bellini (Oxford University NHS Trust); Hamdi Ebdewi, Mohamed Elshaer, Gianpiero Gravante, Benjamin Drake (Kettering General Hospital NHS Foundation Trust); Arikoge Ogedegbe, Dipankar Mukherjee, Chanpreet Arhi, Lola Giwa Nusrat Iqbal (Barking, Havering and Redbridge University Hospitals NHS Trust); Nicholas F Watson, Smeer Kumar Aggarwal, Philippa Orchard, Eduardo Villatoro (Kings Mill Hospital); Peter D Willson, Kam Wa Jessica Mok, Thomas Woodman, Jean Deguara (Kingston Hospital NHS Foundation Trust); Giuseppe Garcea, Benoy I Babu, AR Dennison, Deep Malde, David Lloyd, Steve Satheesan, Omer Al-Taan, Alexander Boddy (University Hospitals of Leicester NHS Trust); John P Slavin, Robert P Jones, Laura Ballance, Stratos Gerakopoulos (Leighton Hospital, Mid Cheshire Hospitals NHS Foundation Trust); Periyathambi Jambulingam, Sami Mansour, Naomi Sakai, Vikas Acharya (Luton & Dunstable University Hospital NHS Foundation Trust); Mohammed M Sadat, Lawen Karim, David Larkin, Khalid Amin (Macclesfield District General Hospital); Amarah Khan, Jennifer Law, Saurabh Jamdar, Stella R Smith, Keerthika Sampat, Kathryn M O'shea (Central Manchester NHS Foundation Trust); Mangta Manu, Fotini M Asprou, Nabeela S Malik, Jessica Chang, Marianne Johnstone (Royal Wolverhampton Hospitals NHS Trust); Michael Lewis, Geoffrey P Roberts, Babu Karavadra, Evangelos Photi (Norfolk and Norwich University Hospitals NHS Foundation Trust); James Hewes, Laura Gould, Alice Chambers, Dan Rodriguez (North Bristol NHS Trust); Derek A O'Reilly, Anthony J Rate, Hema Sekhar, Lucy T Henderson, Benjamin Z Starmer, Peter O Coe, Sotonye Tolofari, Jenifer Barrie (Pennine Acute NHS Trust); Gareth Bashir, Jake Sloane, Suroosh Madanipour, Constantine Halkias, Alexander EJ Trevatt (North Middlesex Trust); David W Borowski, Jane Hornsby, Michael J Courtney, Suvi Virupaksha (North Tees and Hartlepool NHS Foundation Trust); Keith Seymour, Sarah Robinson, Helen Hawkins, Sadiq Bawa, Paul V Gallagher, Alistair Reid, Peter Wood (Northumbria Healthcare NHS Foundation Trust); JG Finch, J Guy Finch, J Parmar, E Stirland (Northampton General Hospital NHS Trust); James Gardner-Thorpe, Ahmed Al-Muhktar, Mark Peterson, Ali Majeed (Sheffield Teaching Hospitals NHS Foundation Trust); Farrukh M Bajwa, Jack Martin, Alfred Choy, Andrew Tsang (Peterborough City Hospital); Naresh Pore, David R Andrew, Waleed Al-Khyatt, Christopher Taylor Santosh Bhandari, Adam Chambers, Dhivya Subramanium (United Lincolnshire Hospitals NHS Trust); Simon K C Toh, Nicholas C Carter, Sophie Tate, Belinda Pearce, Denise Wainwright, Stuart J Mercer, Benjamin Knight (Portsmouth Hospitals NHS Trust); Vardhini Vijay, Swethan Alagaratnam, Sidhartha Sinha, Shahab Khan (The Princess Alexandra Hospital NHS Trust); Shamsi S El-Hasani, Abdulzahra A Hussain (Kings College Hospital NHS Foundation Trust); Vish Bhattacharya, Nisheeth Kansal, Tani Fasih, Claire Jackson (Gateshead Health NHS Foundation Trust); Midhat N Siddiqui, Imran A Chishti, Imogen J Fordham, Zohaib Siddiqui (Lewisham and Greenwich NHS Trust); Harald Bausbacher, Ileana Geogloma, Kabita Gurung (Queen Elizabeth Hospital NHS Trust); George Tsavellas, Pradeep Basynat, Ashish Kiran Shrestha, Sanjoy Basu, Alok Chhabra Mohan Harilingam, Mohamed Rabie, Mansoor Akhtar (East Kent Hospitals University NHS Foundation Trust); Pradeep Kumar, Sadaf F Jafferbhoy, Najam Hussain, Soulat Raza (Burton Hospitals NHS Foundation Trust); Manzarul Haque, Imran Alam, Rabiya Aseem, Shakira Patel, Mehek Asad (Royal Albert Edward Infirmary, Wigan Wrightington and Leigh NHS Trust); Michael I Booth, William R Ball, Christopher PJ Wood, Ana C Pinho-Gomes (Royal Berkshire NHS Foundation Trust); Ambareen Kausar, Moh'd Rami Obeidallah (East Lancashire Hospital Trust); Joseph Varghase, Joshil Lodhia, Donal Bradley, Carla Rengifo, David Lindsay (Royal Bolton Hospital NHS Foundation Trust); Sivakumar Gopalswamy, Ian Finlay, Stacy Wardle, Naomi Bullen (Royal Cornwall NHS Trust); Syed Yusuf Iftikhar, Altaf Awan, Javed Ahmed, Paul Leeder (Royal Derby NHS Foundation Trust); Guiseppe Fusai, Giles Bond-Smith, Alicja Psica, Yogesh Puri (Royal Free, London); David Hou, Fergus Noble, Karoly Szentpali, Jack Broadhurst (Hampshire Hospital NHS Foundation Trust); Ravindra Date, Martin R Hossack, Yan Li Goh, Paul Turner, Vinutha Shetty (Lancashire Teaching Hospitals NHS Foundation Trust); Manel Riera, Christina A W Macano, Anisha Sukha (Royal Shrewsbury Hospital); Shaun R Preston, Jennifer R Hoban, Daniel J Puntis, Sophie V Williams (Royal Surrey County Hospital NHS Foundation Trust); Richard Krysztopik, James Kynaston, Jeremy Batt, Matthew Doe (Royal United Hospital Bath NHS Trust); Andrzej Goscimski, Gareth H Jones, Stella R Smith, Claire Hall (Salford Royal NHS Foundation Trust); Nick Carty, Jamil Ahmed, Sofoklis Panteleimonitis (Salisbury Hospital Foundation Trust); Rohan T Gunasekera, Andrea RG Sheel, Hannah Lennon, Caroline Hindley (Southport and Ormskirk Hospital NHS Trust); Marcus Reddy, Ross Kenny, Natalie Elkheir, Emma R McGlone (St George's Healthcare NHS Trust); Rajasundaram Rajaganeshan, Kate Hancorn, Anita Hargreaves (St Helens and Knowsley Teaching Hospitals NHS Trust); Raj Prasad, David A Longbotham, Dhakshinamoorthy Vijayanand, Imeshi Wijetunga (Leeds Teaching Hospitals); Paul Ziprin, Christopher R Nicolay, Geoffrey Yeldham, Edward Read (Imperial College Healthcare NHS Trust); James A Gossage, Rachel C Rolph, Husam Ebied, Manraj Phull (St Thomas' Hospital, London); Mohammad A Khan, Matthew Popplewell, Dimitrios Kyriakidis, Anwar Hussain (Mid Staffordshire NHS Foundation Trust); Natasha Henley, Jessica R Packer, Laura Derbyshire, Jonathan Porter (Stockport NHS Foundation Trust); Shaun Appleton, Marwan Farouk, Melvinder Basra (Bucks Healthcare NHS Trust); Neil A Jennings, Shahda Ali, Venkatesh Kanakala (City Hospitals Sunderland NHS Foundation Trust); Haythem Ali, Risha Lane, Richard Dickson-Lowe, Prizzi Zarsadias (Tunbridge Wells and Maidstone NHS Trust); Darius Mirza, Sonia Puig, Khalid Al Amari, Deepak Vijayan, Robert Sutcliffe, Ravi Marudanayagam (University Hospital Birmingham NHS Foundation Trust); Zayed Hamady, Abheesh R Prasad, Abhilasha Patel (University Hospital Coventry and Warwickshire NHS Trust); Damien Durkin, Parminder Kaur, Laura Bowen (University Hospital of North Staffordshire NHS Trust); James P Byrne, Katherine L Pearson, Theo G Delisle, James Davies (University Hospital Southampton NHS Foundation Trust); Mark A Tomlinson, Michelle A Johnpulle, Corinna Slawinski (University Hospitals of Morecambe Bay); Andrew Macdonald, James Nicholson, Katy Newton, James Mbuvi (University Hospital South Manchester NHS Foundation Trust); Ansar Farooq, Bhavani Sidhartha Mothe, Zakhi Zafrani, Daniel Brett (Warrington and Halton Hospitals NHS Trust); James Francombe, Philip Spreadborough, James Barnes, Melanie Cheung (South Warwickshire NHS Foundation Trust); Ahmed Z Al-Bahrani, Giuseppe Preziosi, Tomas Urbonas (Watford General Hospital); Justin Alberts, Mekhlola Mallik, Krashna Patel, Ashvina Segaran, Triantafyllos Doulias (West Suffolk NHS Trust); Pratik A Sufi, Caroline Yao, Sarah Pollock (Whittington NHS Trust); Antonio Manzelli, Saj Wajed, Michail Kourkulos, Roberto Pezzuto (Wonford Hospital); Martin Wadley, Emma Hamilton, Shameen Jaunoo, Robert Padwick (Worcestershire Acute Hospitals NHS Trust); Mazin Sayegh, Richard C Newton, Madhusoodhana Hebbar, Sameh F Farag, (Western Sussex Hospitals NHS Foundation Trust); John Spearman, Mohammed F Hamdan, Conrad D'Costa, Christine Blane; (Yeovil District Hospital NHS Trust); Mathew Giles, Mark B Peter, Natalie A Hirst, Tanvir Hossain, Arslan Pannu Yesar El-Dhuwaib, Tamsin E M Morrison, Greg W Taylor (York Teaching Hospital NHS Foundation Trust).

**Northern Ireland** - Ronald LE Thompson, Ken McCune, Paula Loughlin, Roger Lawther (Altnagelvin Area Hospital); Colman K Byrnes, Duncan J Simpson, Abi Mawhinney, Conor Warren (Antrim Area Hospital); Damian McKay, Colin McIlmunn, Serena Martin, Matthew MacArtney (Daisy Hill Hospital); Tom Diamond, Phil Davey, Claire Jones, Joshua M Clements, Ruairi Digney, Wei Ming Chan, Stephen McCain, Sadaf Gull, Adam Janeczko, Emmet Dorrian, Andrew Harris, Suzanne Dawson, Dorothy Johnston, Barry McAree, (Belfast City Hospital, Mater Infirmorum Hospital Belfast and Royal Victoria Hospital); Essam Ghareeb, George Thomas, Martin Connelly, Stephen McKenzie, Krzysztos Cieplucha (South West Acute Hospital); Gary Spence, William Campbell, Gareth Hooks, Neil Bradley (Ulster Hospital).

**Republic of Ireland** – Arnold DK Hill, John T Cassidy, Michael Boland (Beaumont Hospital, Dublin); Paul Burke, Deirdre M Nally (University Hospital Limerick); Arnold DK Hill, Elmoataz Khogali, Wael Shabo, Edrin Iskandar (Louth County Hospital and Our Lady of Lourdes Hospital); Gerry P McEntee, Maeve A O'Neill, Colin Peirce, Emma M Lyons (Mater Hospital, Dublin); Adrian W O'Sullivan, Rohan Thakkar, Paul Carroll, Ivan Ivanovski (Mercy University Hospital); Paul Balfe, Matthew Lee (St Luke's General Hospital Kilkenny);, Des C Winter, Michael E Kelly, Emir Hoti, Donal Maguire; Priyadarssini Karunakaran, Justin G Geoghegan, Frank McDermott, Sean T Martin (St Vincent's University and Private Hospitals, Dublin); Keith S Cross, Fiachra Cooke, Saquib Zeeshan, James O Murphy (Waterford Regional Hospital); Ken Mealy, Helen M Mohan, Yuwaraja Nedujchelyn, Muhammad Fahad Ullah (Wexford General Hospital).

**Scotland -** Irfan Ahmed, Francesco Giovinazzo, James Milburn (Aberdeen Royal Infirmary); Sarah Prince, Eleanor Brooke, Joanna Buchan (Belford Hospital); Ahmed M Khalil, Elizabeth M Vaughan, Michael I Ramage, Roland C Aldridge (Borders General Hospital); Simon Gibson, Gary A Nicholson, David G Vass (Crosshouse Hospital, Ayrshire & Arran); Alan J Grant, David J Holroyd, M Angharad Jones, Cherith MLR Sutton (Dr Gray's Hospital); Patrick O'Dwyer, Frida Nilsson (Gartnavel General Hospital); Beatrix Weber, Tracey K Williamson, Kushik Lalla, Alice Bryant (Gilbert Bain Hospital); C Ross Carter, Craig R Forrest, David I Hunter (Glasgow Royal Infirmary); Ahmad H Nassar, Mavis N Orizu, Katrina Knight, Haitham Qandeel (Monklands Hospital); Stuart Suttie, Rowena Belding, Andrew McClarey (Ninewells Hospital); Alan T Boyd, Graeme JK Guthrie, Pei J Lim, Andreas Luhmann (Perth Royal Infirmary); Angus JM Watson, Colin H Richards, Laura Nicol, Marta Madurska (Raigmore Hospital); Ewen Harrison, Kathryn M Boyce, Amanda Roebuck, Graeme Ferguson (Royal Infirmary of Edinburgh); Pradeep Pati, Michael S J Wilson, Faith Dalgaty, Laura Fothergill (Stracathro Hospital); Peter J Driscoll, Kirsty L Mozolowski, Victoria Banwell, Stephen P Bennett (Victoria Hospital, Kirkcaldy); Paul N Rogers, Brendan L Skelly, Claire L Rutherford, Ahmed K Mirza (Western Infirmary Glasgow).

**Wales** –Taha Lazim, Henry C C Lim, Diana Duke, Talat Ahmed (Bronglais General Hospital); William D Beasley, Marc D Wilkinson, Geta Maharaj, Cathy Malcolm (Glangwili General and Prince Philip Hospital); Timothy H Brown, Bilal Al-Sarireh, Guy M Shingler, Nicholas Mowbray, Rami Radwan (Morriston and Singleton Hospitals); Paul Morcous, Simon Wood, Abbas Kadhim (Princess of Wales Hospital); Duncan J Stewart, Andrew L Baker, Nicola Tanner, Hrishikesh Shenoy (Wrexham Maelor Hospital).

**Data validators -** Shazia Hafiz, Joshua A. De Marchi, Deepak Singh-Ranger, Elzanati Hisham, Paul Ainley, Stephen O'Neill. John Terrace, Sara Napetti, Benjamin Hopwood, Thomas Rhys, Justine Downing, Sam Kanavati, Maria Coats, Danail Aleksandrov, Charlotte Kallaway, Salama Yahya, Beatrix Weber, Alexa Templeton, Martin Trotter, Christina Lo, Ajit Dhillon, Nick Heywood, Yousif Aawsaj, Alhafidz Hamdan, Obuobi Reece-Bolton, Andrew McGuigan, Yousef Shahin, Aymon, Ali Alison Luther, James A Nicholson, Ilayaraja Rajendran, Matthew Boal, Judith Ritchie.
